# Case Report: Impella CP-protected percutaneous coronary intervention of the last remaining vessel—a LIMA-to-LAD graft—complicated by no-reflow and rescued with a drug-coated balloon

**DOI:** 10.3389/fcvm.2026.1951936

**Published:** 2026-10-21

**Authors:** Fotios Toulgaridis, Stavros Tzortzis, Nikolaos Tsiamis, Konstantinos Sassalos, Nikolaos Karanikolas, Ioannis Mavrogenis, Anastasia Kitsiou, Georgios Triantis

**Affiliations:** 1 Department of Interventional Cardiology, Catheterization Laboratory, General Hospital of Attica ’Sismanoglio–Amalia Fleming’, Athens, Greece; 2 Department of Cardiology, General Hospital of Attica ’Sismanoglio’, Athens, Greece

**Keywords:** drug-coated balloon, guide extension catheter, Impella CP, last remaining vessel, left internal mammary artery graft, no-reflow, percutaneous mechanical circulatory support, protected PCI

## Abstract

Percutaneous coronary intervention (PCI) of the last remaining conduit in a patient with severely impaired left ventricular function is among the most hazardous procedures in contemporary interventional cardiology, as any interruption of flow essentially constitutes a global ischemic insult. We describe a 77-year-old man, a former heavy smoker with an 80-pack-year smoking history, who had undergone three-vessel coronary artery bypass grafting (CABG) 22 years previously. His medical history also included chronic obstructive pulmonary disease, severe heart failure with a left ventricular ejection fraction of 25–30%, and severe mitral regurgitation (MR). At presentation, he had Canadian Cardiovascular Society class III angina and New York Heart Association class III symptoms. The most recent coronary angiogram, performed 6 months earlier, demonstrated chronic total occlusion (CTO) of both saphenous vein grafts, in addition to CTO of all three native epicardial coronary arteries. Thus, the left internal mammary artery (LIMA) graft to the left anterior descending artery (LAD) represented the sole functional conduit, directly perfusing the LAD territory and supplying the right coronary and circumflex territories through primarily septal and epicardial collaterals, respectively. The LAD displayed two sequential severe stenoses (≥80% each), one at the distal LIMA–LAD anastomosis and another further distally in the native vessel. In accordance with current coronary revascularization guidelines, the Heart Team recommended evaluation by cardiac surgeons. Two independent cardiothoracic surgeons declined redo CABG as a viable therapeutic option. Complex and high-risk indicated PCI (CHIP) was therefore considered the only feasible revascularization strategy. Additional hemodynamic support with percutaneous mechanical circulatory support (pMCS) was deemed necessary. Residual MR and arrhythmic risk, including the potential indication for an implantable cardioverter-defibrillator (ICD), were planned to be reassessed 3 months after successful percutaneous revascularization. CHIP was performed under full hemodynamic support with a continuous-flow microaxial pump (Impella CP with SmartAssist) inserted via the left femoral artery, while coronary intervention was performed through the ipsilateral radial artery using a 6-F IMA guiding catheter. The lesions were successfully crossed with a workhorse wire, despite substantial technical difficulty and with the aid of a microcatheter; however, distal flow was immediately lost, and intracoronary nitroglycerin failed to restore it. Balloon angioplasty restored flow only partially, and a decision was made to proceed with stent implantation. However, stent delivery proved challenging, requiring a guide-extension catheter to be advanced deep into the LIMA body using the so-called ‘inchworming technique.’ Despite successful implantation of the first drug-eluting stent (DES) at the distal anastomotic lesion, a second DES could not be advanced to the more distal native LAD lesion, despite the use of multiple guidewires and supportive guiding-catheter strategies. Therefore, a drug-coated balloon (DCB) was used as a bailout strategy, immediately restoring TIMI grade 3 flow and leaving only a minor, non-flow-limiting type B dissection. Impella support was discontinued early because of access-related left lower-limb ischemia, and successful percutaneous hemostasis was achieved using a suture-based closure technique. Peak high-sensitivity troponin I, measured the following evening, was 10,800 pg/mL. The patient was discharged ambulatory on day 2 and remained asymptomatic at 3-month follow-up, when an ICD was implanted for primary prevention. This case illustrates the critical role of pMCS in maintaining hemodynamic stability when native coronary flow is lost during PCI of the last remaining conduit and highlights the value of DCB angioplasty as a bailout strategy when stent delivery is not feasible.

## 1 Introduction

The concept of complex and high-risk indicated PCI (CHIP) describes patients in whom significant comorbidity, adverse hemodynamics and technically demanding coronary anatomy converge, and for whom revascularization is nonetheless indicated (1–3). Within this population, intervention on the last remaining vessel occupies a category of its own. When a single conduit supplies the entire myocardium of a poorly contracting ventricle, the usual reassurance of interventional practice — that a transient occlusion is regionally tolerated — no longer holds. Loss of flow becomes a global event, and the interval between an uneventful case and refractory cardiogenic shock may be measured in seconds.

It is precisely this anatomy that defined eligibility in PROTECT II, the randomized comparison of the Impella 2.5 microaxial pump against intra-aortic balloon counterpulsation in non-emergent high-risk PCI: unprotected left main or last patent coronary vessel with a left ventricular ejection fraction (LVEF) ≤ 35%, or three-vessel disease with LVEF <30% (4).

Patients with remote coronary artery bypass grafting (CABG) add a further layer of difficulty. Progressive graft attrition leaves the surviving arterial conduit carrying a perfusion burden far beyond its original territory, often filling other territories retrogradely. Intervention at the distal anastomosis of an internal mammary graft is notoriously demanding: guide support is limited, the conduit is long, tortuous and prone to spasm and pseudolesion formation, and equipment delivery frequently fails where lesion crossing has succeeded (5, 6).

We describe a case combining all these features, in which the intervention did not proceed smoothly — no-reflow developed after wiring, a second stent proved undeliverable, and the definitive treatment of the distal lesion was a bailout drug-coated balloon (DCB). We report it in accordance with the CARE guidelines because the difficulties encountered, and the strategies that resolved them, carry more instructional value than an uncomplicated result would.

## 2 Case description

### 2.1 Patient information

A 77-year-old man presented with a six-month history of increasingly frequent angina on minimal exertion (Canadian Cardiovascular Society [CCS] class III) together with symptoms and signs of heart failure (New York Heart Association [NYHA] class III).

His history included an anterior non-ST-elevation myocardial infarction followed by triple-vessel CABG performed 22 years earlier: LIMA to the LAD, a saphenous vein graft (SVG) to the right coronary artery (RCA), and an SVG to an obtuse marginal branch. Both vein grafts had been known to be occluded for the preceding ten years, and all three native epicardial vessels — LAD, left circumflex (LCx) and RCA — were chronically totally occluded. He also suffered from arterial hypertension, dyslipidemia and chronic obstructive pulmonary disease, since he was a heavy prior smoker (80-pack-year smoking history).

### 2.2 Diagnostic assessment

Transthoracic echocardiography demonstrated a left ventricular ejection fraction (LVEF) of 25%–30% with diffuse hypokinesia. The examination confirmed the presence of severe functional mitral regurgitation, alongside moderate tricuspid regurgitation, and an estimated pulmonary artery systolic pressure (PASP) of 40 mmHg.

Coronary angiography, performed at another center 6 months earlier, demonstrated total occlusion of the RCA and LCx, as well as their respective saphenous vein grafts; severe left main coronary artery (LM) stenosis, with the LM supplying a large first diagonal branch and the LAD occluded immediately distal to its origin; and a patent LIMA-to-LAD graft with two sequential severe stenoses—one at the distal LIMA-to-LAD anastomosis and another more distally in the native LAD at the bifurcation with the second diagonal branch. The LIMA-to-LAD graft supplied collateral flow to both the RCA and LCx territories and constituted the sole remaining functional conduit supplying the entire myocardium (Figures 1, 2).

**Figure 1:**
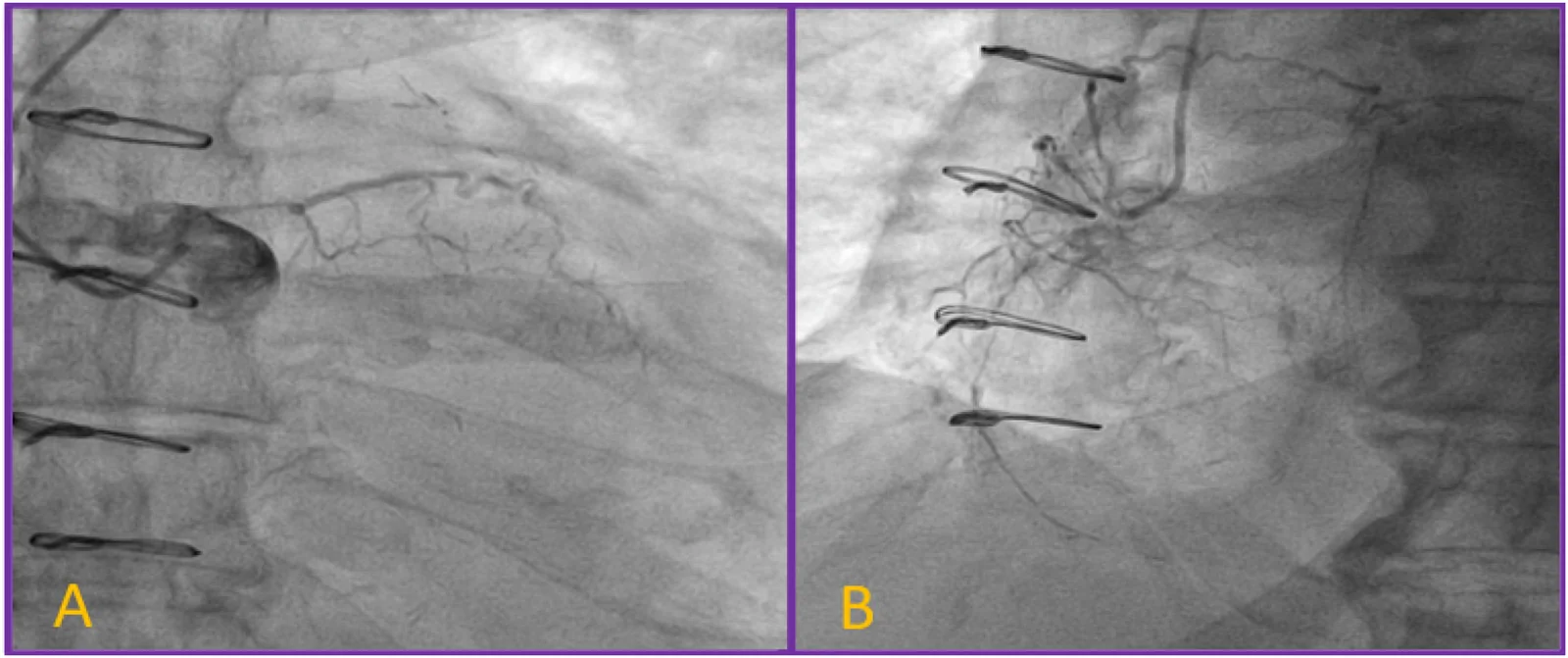
Native 3-vessel coronary artery disease **(A)** Nonselective left coronary artery cannulation (AP/caudal projection) demonstrating severe diffuse LM coronary artery stenosis, a mid-LAD CTO with ambiguous cap distal to the origin of a large septal branch, and a proximal-lcx CTO distal to the origin of the 1st OM branch-again with an ambiguous proximal cap as well. **(B)** Selective right coronary angiography (RAO 25° projection) demonstrating severe diffuse RCA disease and a mid-RCA CTO, with a distinct microchannel following the course of the occluded vessel distal to the origin of the 1st acute marginal branch. An epicardial collateral connection between the conus branch and the proximal LAD, forming Vieussens’ arterial ring, is also visible.

**Figure 2:**
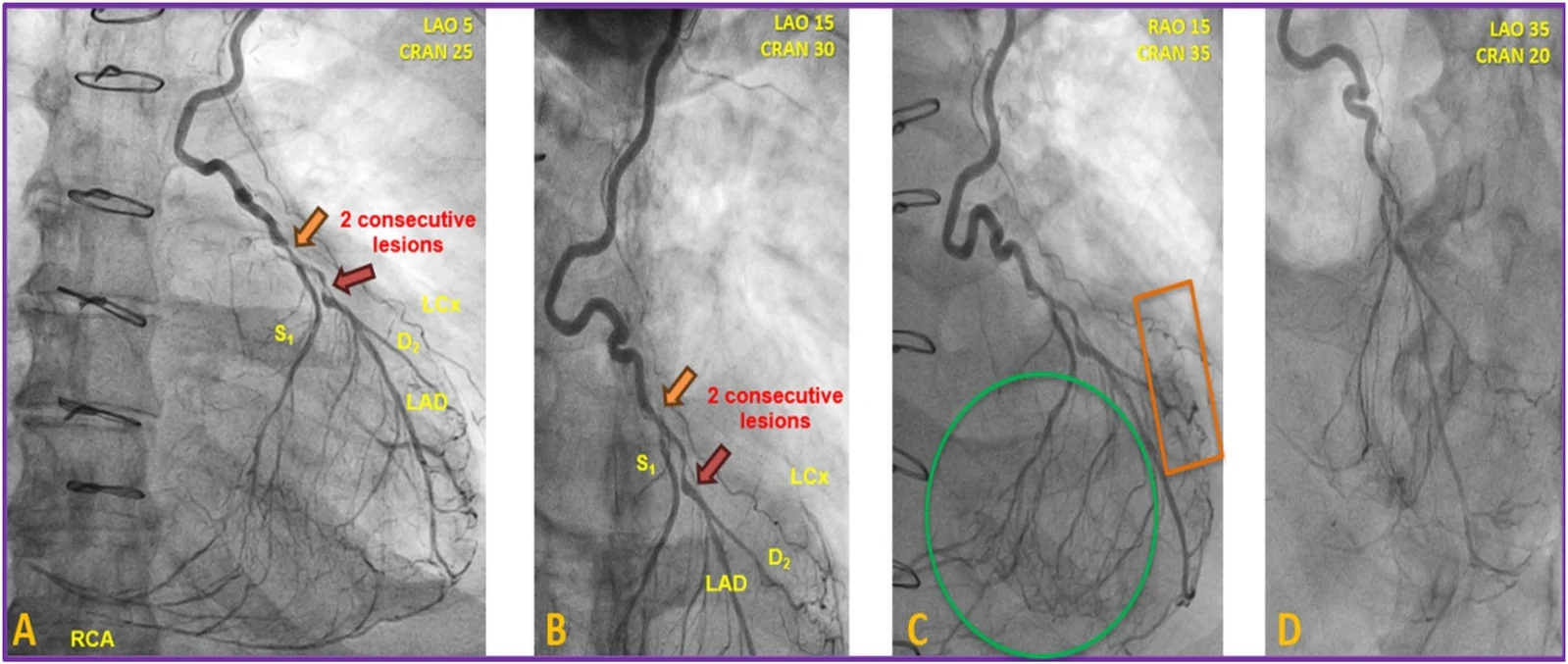
Antegrade LIMA–LAD filling and its collateral supply to RCA and lcx. **(A,B)** Selective LIMA injection [**(A)** LAO 5°/Cranial 25° projection and **(B)** LAO 15°/Cranial 30°] showing the last-remaining patent LIMA graft with two sequential severe stenoses (each≥80%) at the distal anastomosis (orange arrow) and further distally (red arrow). *Arrows indicate the target lesions.* **(C)** Selective LIMA injection (RAO 15°/Cranial 35° projection). Retrograde collateral filling of the left circumflex artery (orange box) and the right coronary artery (green circle) from the patent LIMA graft. Notice how both the inferior wall (RCA territory) and the lateral wall (LCx territory) are essentially retrogradely supplied by the patent LIMA-LAD system. **(D)** Selective LIMA angiography (LAO 35°/cranial 20° projection), providing an overall assessment of the native epicardial coronary circulation through the LIMA–LAD system.

### 2.3 Heart team assessment

Redo surgical revascularization was declined by two independent cardiothoracic surgeons on the grounds of prohibitive operative risk — reduced LVEF, prior sternotomy, and chronic obstructive pulmonary disease. With medical therapy failing to control symptoms, PCI was considered the only feasible revascularization option, and the two sequential stenoses of the LIMA–LAD system were selected as the treatment targets. Importantly, the RCA territory was dependent on LAD-derived collaterals; therefore, improving LAD flow was also expected to enhance collateral perfusion of the RCA territory. In our view, viability assessment of the RCA or LCx territories would not have altered the revascularization strategy, as the patient's CCS class III angina was considered primarily attributable to the tandem LAD lesions.

This situation — severe left ventricular dysfunction with complex coronary disease and surgical ineligibility established by Heart Team evaluation — is explicitly addressed by contemporary European guidance (7). Given that occlusion of this conduit at any point during the procedure would abolish perfusion to the whole left ventricle, the procedure was planned from the outset as a protected intervention with pre-emptive percutaneous mechanical circulatory support (pMCS), consistent with expert consensus recommendations (2, 8).

### 2.4 Procedure

Pre-procedural computed tomography confirmed a common femoral and iliac luminal diameter exceeding 6 mm despite mild-to-moderate iliac stenosis, and the femoral artery was consequently judged suitable for large-bore access. An Impella CP device with SmartAssist was introduced through a 14-F left femoral sheath. A 6-F IMA guiding catheter was advanced from the left radial artery and engaged in the LIMA ostium.

Full hemodynamic support was established with the Impella CP prior to any coronary manipulation. The Impella device provided mechanical circulatory support for a total duration of 2 h. During this period, it was maintained at a performance level of P9, achieving a peak supported flow of 3.8 L/min. Procedural anticoagulation was managed with the administration of unfractionated heparin according to standard acute clotting time (ACT) values (≥250 s) (Figure 3).

**Figure 3:**
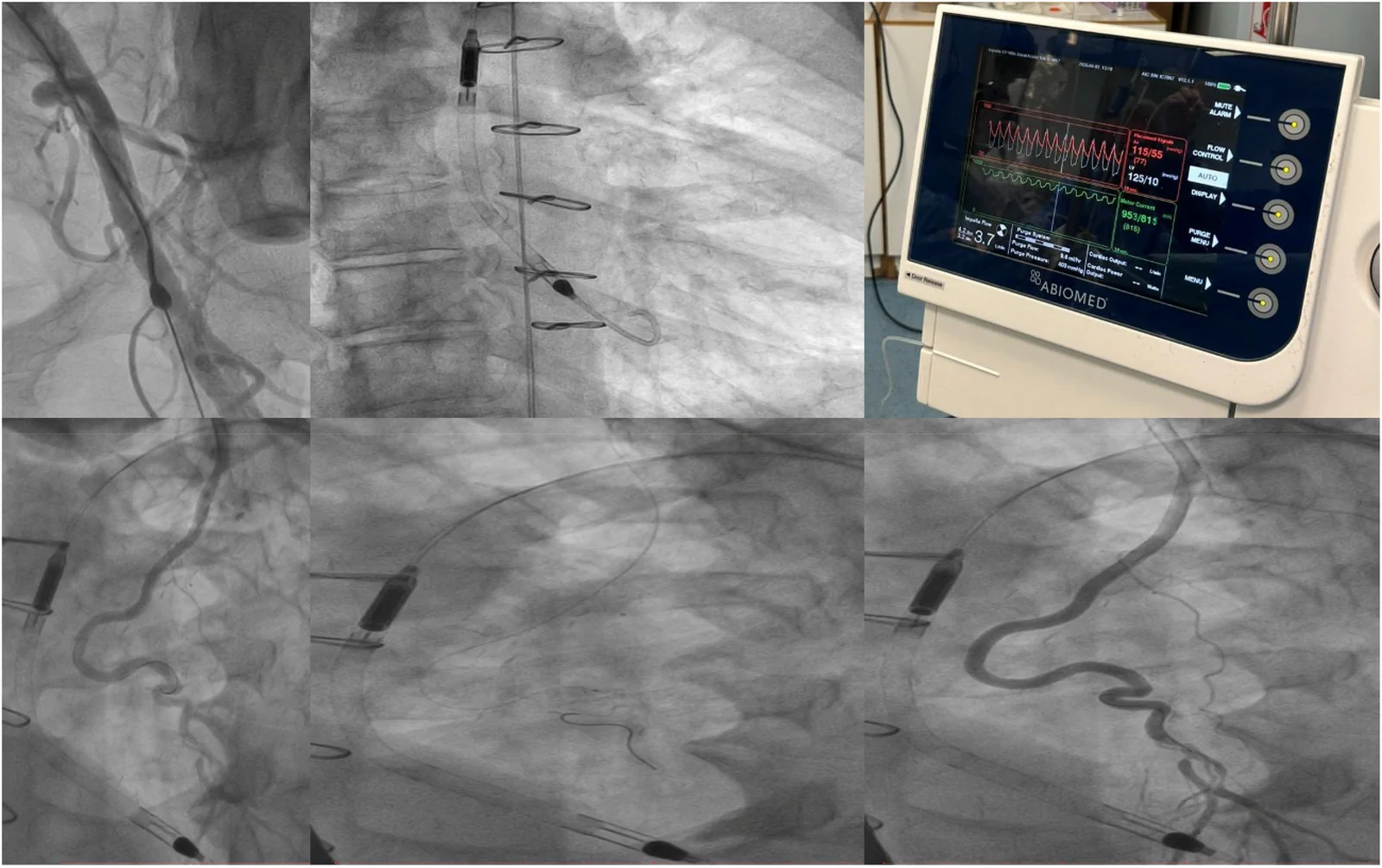
Hemodynamic support. Impella CP in position across the aortic valve with the corresponding SmartAssist placement signal and motor current waveforms, alongside the invasive arterial pressure trace during the period of no-reflow, illustrating maintenance of systemic perfusion despite absent antegrade coronary flow.

Despite multiple guidewire strategies—including workhorse, hydrophilic-tip, hybrid, and polymer-jacketed wires—the second stenosis remained uncrossable, partly because of marked vessel angulation and the presence of a bifurcation lesion. The target lesions were finally crossed successfully using a Sion guidewire supported by a Corsair Pro XS microcatheter in a two-step fashion. Before successful lesion crossing, the Sion guidewire was inadvertently advanced into the second diagonal branch at the bifurcation, providing sufficient support to advance the Corsair Pro XS microcatheter to this level. Selective contrast injection through the microcatheter confirmed its position within the diagonal branch, excluding any possible wire-induced dissections. The microcatheter was subsequently repositioned to redirect the guidewire trajectory toward the distal LAD. This side-branch anchoring maneuver provided additional support and improved alignment, ultimately facilitating successful lesion crossing with the Sion guidewire supported by the Corsair Pro XS microcatheter (Figure 4).

**Figure 4:**
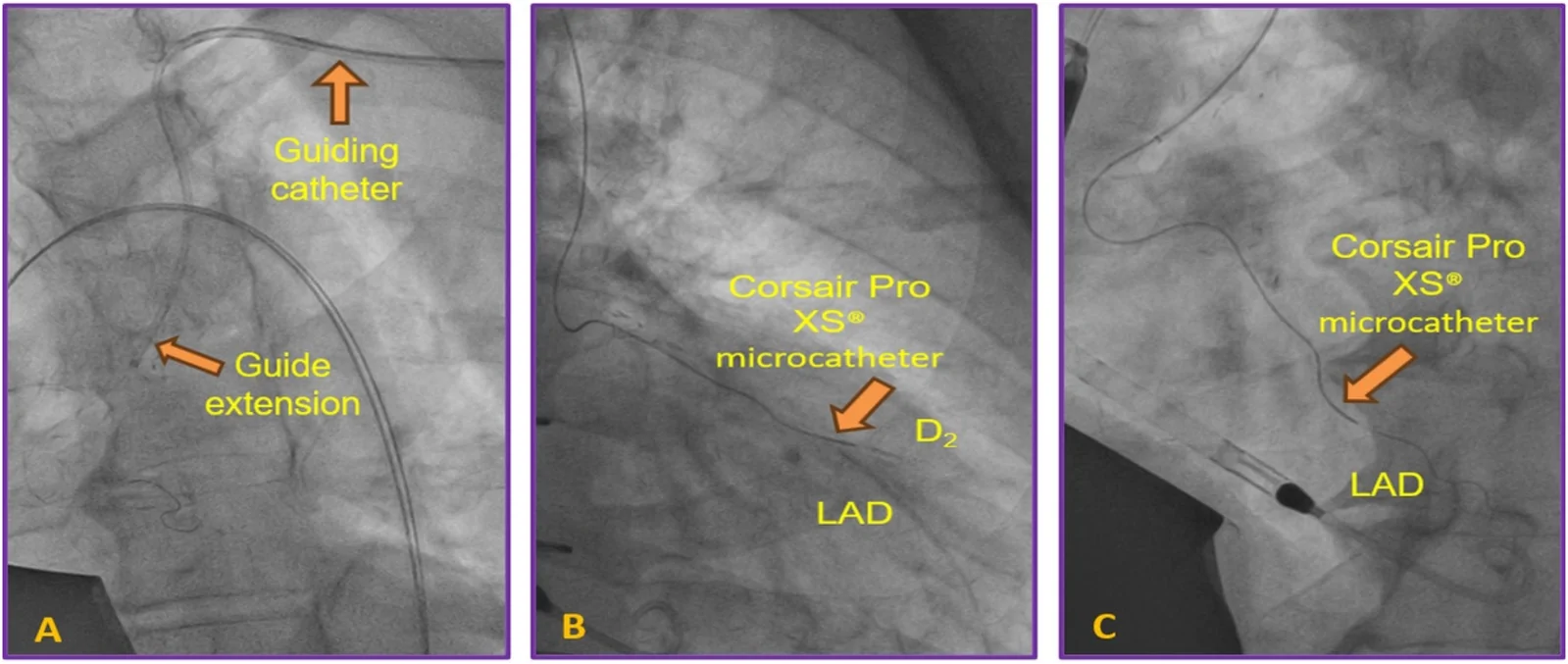
Procedural sequence. **(A)** A 6-F IMA guiding catheter and a 6-F Guidezilla guide-extension catheter, with the latter advanced into the distal third of the LIMA using the inchworming technique. **(B)** Initial attempts to cross into the LAD with multiple guidewires were unsuccessful. A 0.014-inch Sion workhorse guidewire was eventually advanced into the 2nd diagonal branch through a Corsair Pro XS microcatheter. Contrast injection through the microcatheter tip confirmed the guidewire position. **(C)** Redirection of the Corsair Pro XS microcatheter facilitated successful guidewire advancement into the distal LAD.

However, further technical challenges arose. Immediately after guidewire passage into the distal LAD, contrast opacification was lost from the distal LIMA–LAD anastomosis and beyond. Given that the activated clotting time (ACT) was within the therapeutic range, the differential diagnosis included a LIMA pseudolesion, wire-induced spasm, or iatrogenic dissection of the LIMA–LAD system. Intracoronary nitroglycerin failed to restore flow, and additional vasodilator therapy was similarly ineffective. Gentle guidewire pullback also failed to improve distal perfusion, raising concern for an underlying dissection. To restore distal flow and improve myocardial perfusion, balloon angioplasty was performed because no clearly defined landing zone for stent implantation was available. This approach was also intended to facilitate subsequent stent delivery and implantation.

Balloon angioplasty was subsequently performed using 2.0 × 15-mm and 2.5 × 15-mm semi-compliant (SC) balloons, followed by inflation of a 2.5 × 15-mm non-compliant (NC) balloon at 18 atm. This restored TIMI grade 2 flow without evidence of a flow-limiting dissection, indirectly suggesting that the preceding acute flow impairment may have been related to a pronounced concertina effect during device manipulation. At this stage, subsequent stent implantation was considered a reasonable strategy. Initially, a two-stent PCI strategy was selected, as delivery and implantation of a single long stent through the LIMA and across both tandem lesions was considered technically unfavorable. However, despite adequate lesion preparation, the stents could not initially be advanced to the target lesions, indicating a deliverability rather than a lesion-crossing failure. Additional guide support was considered necessary to facilitate stent delivery. A 6-F Guidezilla guide-extension catheter was therefore employed and advanced into the distal third of the LIMA using the so-called ‘inchworming technique,’ facilitated by a 2.5 × 15-mm SC balloon (Figure 5).

**Figure 5:**
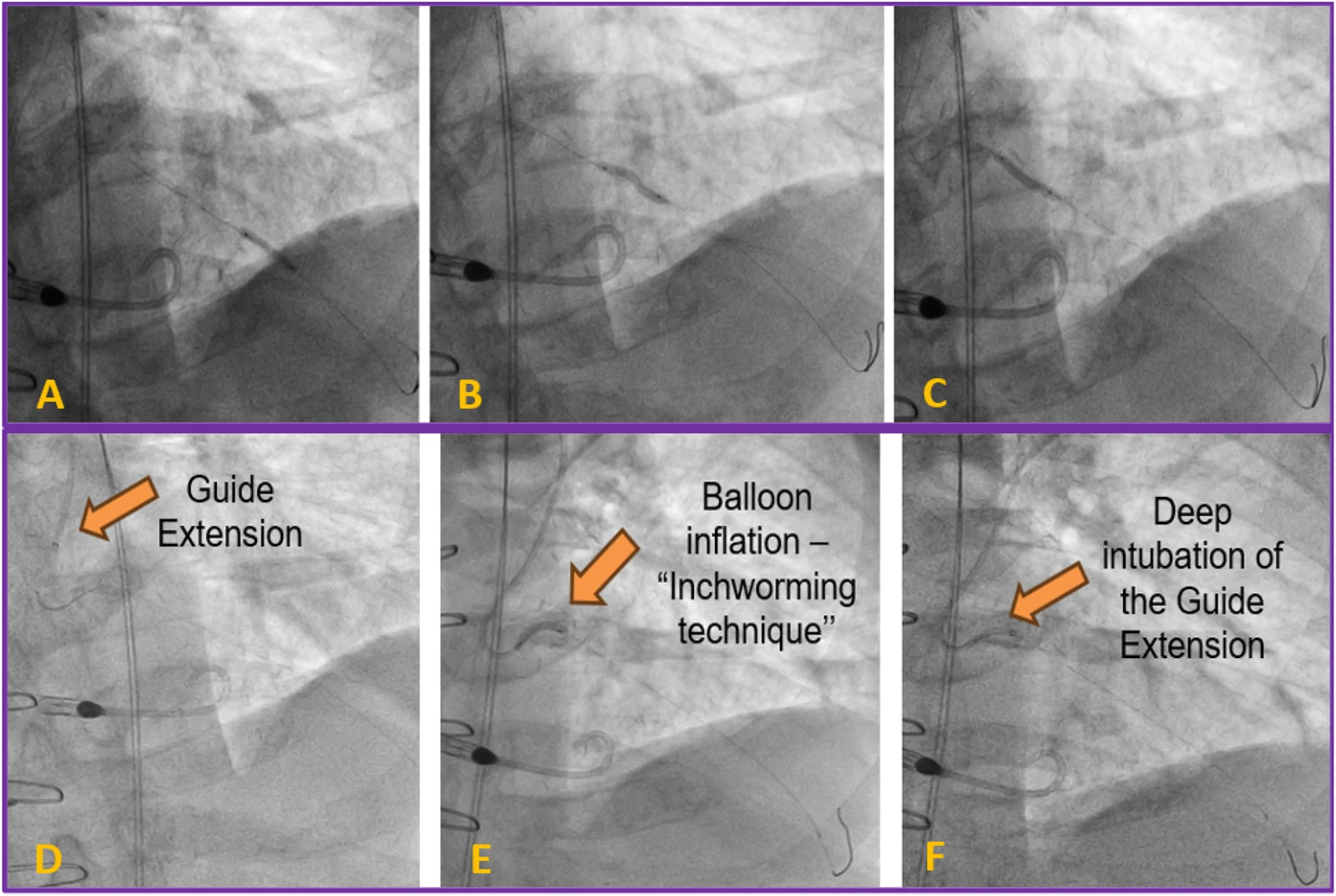
Steps of complex and higH–risk indicated PCI. **(A–C)** Lesion Preparation: Both lesions were predilated using 2.00 × 15 mm and 2.50 × 15 mm semi-compliant balloons, followed by dilation with a 2.50 × 15 mm non-compliant balloon at 18 atm. **(D–F)** Stent-delivery difficulty: Despite adequate lesion preparation, the drug-eluting stents could not be advanced across the lesions. A 6-F Guidezilla guide-extension catheter was therefore advanced into the distal third of the LIMA using the inchworming technique to provide additional support.

Although stent implantation is generally performed in a distal-to-proximal sequence in our catheterization laboratory, the preceding episode of acute LIMA flow compromise and the marked susceptibility of this tortuous conduit to the accordion phenomenon prompted a modification of this strategy. We therefore elected to implant the proximal DES first, thereby creating a stable scaffold that could facilitate subsequent guide-extension advancement and provide additional support during treatment of the distal lesion. Consequently, the extra support provided by deeper Guidezilla advancement enabled the deployment of a 2.5 × 16-mm Firehawk Liberty DES at the distal LIMA–LAD anastomosis at 20 atm. Nevertheless, delivery of a second stent to the more distal LAD lesion proved unsuccessful despite the use of multiple buddy wires and supportive catheter strategies.

Subsequent angiography, however, demonstrated loss of antegrade flow beyond the first DES, raising suspicion of a distal stent-edge dissection. Angiographically, the appearance was consistent with an advanced iatrogenic coronary dissection. Intracoronary imaging to verify guidewire position within the true lumen was considered unnecessary, as guidewire position had been maintained throughout the procedure, making loss of true-lumen access unlikely. Importantly, immediate restoration of coronary flow was the primary procedural objective. Although DES implantation would generally be the preferred treatment for a severe iatrogenic coronary dissection, further stent delivery was not feasible because of the marked deliverability limitations. An alternative strategy was thus required. Given its lower device profile compared with a DES, a drug-coated balloon (DCB) was considered more deliverable to the distal target lesion. At this stage, the procedure was completed using a hybrid DES–DCB strategy. The Impella CP provided effective hemodynamic support throughout this prolonged period of compromised coronary flow, allowing the intervention to proceed safely.

Prior to DCB angioplasty, adequate lesion preparation was performed to further optimize luminal gain using a 2.5 × 15-mm NC balloon inflated at 18 atm with prolonged inflation. Subsequently, a 2.5 × 15-mm paclitaxel-coated balloon was inflated at 12 atm for 60 s without complications. Final angiography demonstrated no residual stenosis and a TIMI grade 3 flow, with only a minor, non-flow-limiting type B dissection, which was managed conservatively (Figure 6).

**Figure 6:**
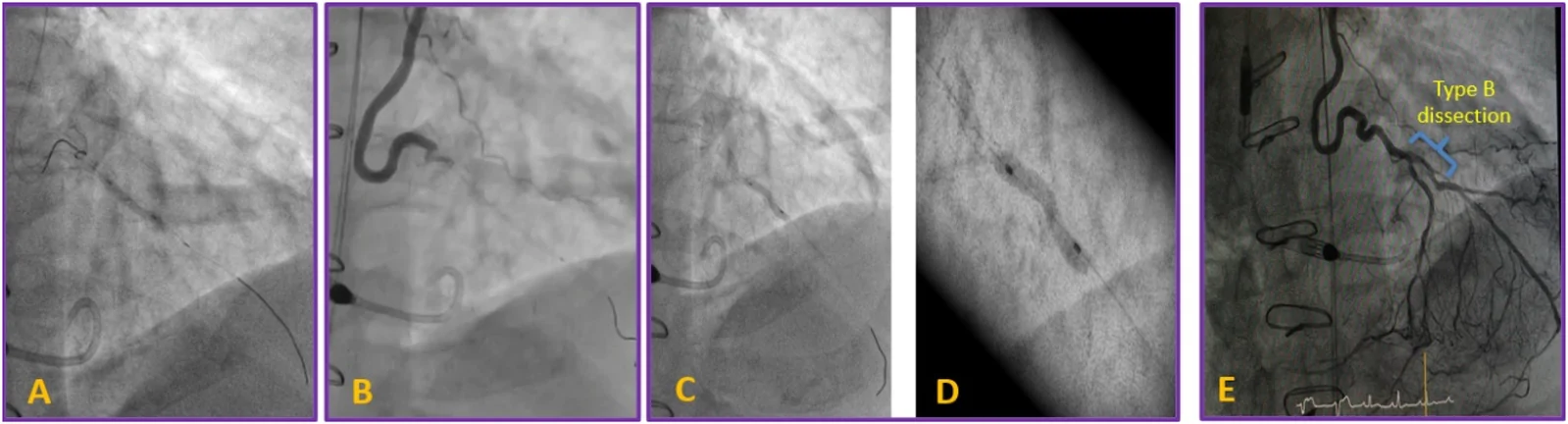
Initial DES implantation, subsequent no-reflow, and successful bailout management with DCB angioplasty, as demonstrated by final angiography. **(A)** Deployment of a 2.50 × 16 mm Firehawk Liberty drug-eluting stent (DES) at 20 atm across the LIMA–LAD anastomosis. **(B)** No-reflow following DES implantation; intracoronary nitroglycerin failed to restore distal coronary flow. **(C**,**D)** Because no-reflow persisted and delivery of a second DES into the distal native LAD was considered technically challenging, bailout balloon angioplasty was performed. Using a buddy-wire technique, the distal lesion was successfully treated with a 2.50 × 15 mm paclitaxel-coated balloon inflated at 12 atm, with overlap at the distal edge of the proximally implanted DES, constituting a hybrid DES–DCB strategy. **(E)** Final angiography demonstrating restoration of TIMI grade 3 flow and a minor, non-flow-limiting type B dissection at the DCB-treated segment (blue brace).

The patient experienced only minimal chest discomfort, despite transient ST-segment depression on ECG during procedural manipulations. Approximately 20 min before completion of the procedure, he developed left lower-extremity pain and clinical signs of limb ischemia, attributed to the 14-F femoral sheath in the setting of pre-existing peripheral arterial disease. As he remained hemodynamically stable, Impella support was promptly discontinued and the device was removed. Percutaneous arteriotomy closure was achieved, using two Perclose ProGlide suture-mediated closure devices. A local hematoma extending across the inguinal ligament subsequently developed; however, no major vascular complications occurred, and the limb ischemic symptoms were resolved.

### 2.5 Outcome and follow-up

The patient remained hospitalized for a further 2 days without major complications and was discharged ambulatory. At discharge, he was receiving guideline-directed medical therapy for heart failure and dual antiplatelet therapy with aspirin and clopidogrel, planned for at least 12 months.

At 1- and 3-month follow-up, he remained free of angina and was in NYHA functional class II. At 3 months, an implantable cardioverter-defibrillator was implanted for primary prevention of sudden cardiac death.

Follow-up invasive coronary angiography is planned at 12 months to assess the long-term angiographic outcome of the hybrid DES–DCB strategy and to inform subsequent decisions regarding the duration of dual antiplatelet therapy.

## 3 Discussion

### 3.1 Principal observations

Three features of this case merit emphasis.

First, percutaneous mechanical circulatory support was not a precaution but a prerequisite. In the setting of last-vessel anatomy, the loss of distal flow that occurred immediately after guidewire passage—and persisted despite intracoronary nitroglycerin during lesion preparation and stent implantation—would, in an unsupported patient with an LVEF of 25%–30%, have carried a substantial risk of precipitating cardiogenic shock and necessitating procedural abandonment. Moreover, pharmacological treatment of acute coronary flow impairment is not uniformly effective, and no pharmacological agent can be relied upon to consistently restore flow once severe flow compromise has occurred (9). Continuous-flow support decoupled systemic perfusion from left ventricular performance and allowed the operators to continue working methodically through a prolonged period of absent antegrade coronary flow. The counterfactual cannot be observed, but the sequence of events makes the contribution of support unusually legible.

Second, the limiting technical problem was equipment deliverability, not lesion crossing. The wire and microcatheter reached the distal vessel; the stents would not follow. This distinction is characteristic of intervention in long, tortuous internal mammary conduits and it dictates the solution: rather than escalating lesion preparation indefinitely, the operators changed the support vector by advancing a guide extension deep into the distal third of the graft using an inchworming technique. Although technically challenging, this approach ultimately enabled deployment of the anastomotic stent; however, failure to deliver a second stent to the distal lesion dictated the subsequent procedural strategy.

Third, the DCB functioned as a genuine bailout. When a second stent could not be delivered despite multiple buddy wires and support catheters, the alternatives were to leave the distal lesion untreated in a patient whose entire myocardium depended on it, or to accept a leave-nothing-behind strategy. The final angiographic result, with restoration of TIMI grade 3 flow after DCB angioplasty and only a minor, non-flow-limiting type B coronary dissection, together with the patient's symptom-free status at 3-month follow-up, supports this strategy. Evidence supporting DCB use in *de novo* coronary lesions continues to expand, and this case highlights a further pragmatic application: bailout DCB angioplasty when stent delivery is not feasible because of complex coronary anatomy (10, 11).

### 3.2 Hemodynamic support in high-risk PCI: what the evidence supports

The evidence for elective pMCS in high-risk PCI remains narrower than its adoption. BCIS-1 found no reduction in major adverse cardiac and cerebrovascular events with elective intra-aortic balloon pump insertion before high-risk PCI (12). PROTECT II, the largest randomized comparison of the Impella 2.5 against intra-aortic balloon counterpulsation in non-emergent high-risk PCI, was halted early for futility on its 30-day intention-to-treat endpoint; the Impella arm nonetheless showed superior hemodynamic support, reflected in a smaller maximal fall in cardiac power output, and a per-protocol advantage in major adverse events at 90 days (4). Reanalysis using a validated definition of periprocedural myocardial infarction found lower rates of major adverse events and of major adverse cardiac and cerebrovascular events at 90 days with Impella (13). Registry data have been broadly consistent (14, 15).

The appropriate inference is not that pMCS improves outcomes across high-risk PCI generally, but that it supplies a predictable hemodynamic reserve which is decisive where the mechanism of harm is transient global ischemia. Our patient sat squarely in that category, and the intraprocedural course confirmed it. The corollary is that the decision to deploy support should follow from an explicit prediction of what will happen to the circulation when the target vessel is occluded, rather than from a generic risk score.

### 3.3 The cost of support, and honest accounting

This case also illustrates the price of large-bore support. The 14-F sheath produced lower limb ischemia in a patient with established peripheral arterial disease, notwithstanding a computed tomography assessment that had shown apparently adequate vessel caliber. This acts as a reminder that luminal diameter alone does not predict tolerance of a large sheath, and that the duration of support itself is a risk variable. Recognizing the complication and weaning the device immediately, once hemodynamic stability permitted, was the correct response and avoided escalation.

### 3.4 Take-away lessons

In the contemporary PCI era, increasingly complex coronary anatomy is encountered in routine clinical practice, hence CHIP is gaining ground. Especially in last-vessel anatomy, mechanical circulatory support should be in place before the first wire is advanced, not held in reserve; flow can be lost within seconds of wiring and may not be recoverable pharmacologically. As underscored by our case, Impella CP provided effective hemodynamic support and maintained circulatory stability during loss of coronary flow, allowing successful completion of PCI.

Mastery of guide-extension catheter techniques, including deep intubation and the ‘inchworming technique,’ may be crucial for device delivery through severely tortuous LIMA grafts or angulated vessels.

When stent delivery is not feasible, despite adequate lesion preparation, DCB angioplasty is a legitimate and effective fallback and may provide a valuable bailout strategy in selected complex anatomies.

## 4 Conclusion

Percutaneous revascularization of a last remaining LIMA-to-LAD conduit in a patient with severe left ventricular dysfunction and prohibitive surgical risk was accomplished under continuous-flow mechanical circulatory support, despite intraprocedural no-reflow and the failure of conventional stent delivery. Support maintained systemic perfusion throughout a prolonged period of absent coronary flow; a guide extension resolved deliverability; and a drug-coated balloon rescued the distal lesion. The patient was discharged 48 h after the procedure and remained free of angina at 3-month follow-up. Meticulous pre-procedural planning, pre-emptive hemodynamic support, and a prepared repertoire of bailout strategies are what make such procedures feasible.

## Data Availability

The original contributions presented in the study are included in the article/Supplementary Material, further inquiries can be directed to the corresponding author.
